# Population-level and longitudinal MIC distributions for antimicrobials against drug-resistant *Mycobacterium tuberculosis* in Wuxi, a low-epidemic city in China, from 2019 to 2023

**DOI:** 10.3389/fcimb.2026.1866051

**Published:** 2026-07-08

**Authors:** Song Gao, Fan Tu, Huan Ding, Qi Zhang, Shipeng Zhang, Shiya Shen, Jun Liu, Zhuping Xu

**Affiliations:** 1 Department of Chronic Infectious Disease Control and Prevention, The Affiliated Wuxi Center for Disease Control and Prevention of Nanjing Medical University, Wuxi Center for Disease Control and Prevention, Wuxi, China; 2 Department of Laboratory Medicine, Affiliated Wuxi Fifth Hospital of Jiangnan University, The Fifth People’s Hospital of Wuxi, Wuxi, China

**Keywords:** drug resistance, drug-resistant tuberculosis, MIC, resistance patterns, temporal trends, tuberculosis epidemiology

## Abstract

**Background:**

Drug-resistant tuberculosis (DR-TB) remains a significant global health challenge, with increasing resistance to both first- and second-line anti-tuberculosis drugs. Understanding the demographic distribution, temporal trends, and resistance profiles of DR-TB is essential for improving treatment strategies and public health interventions.

**Objective:**

This study aimed to examine the demographic characteristics, temporal distribution, and minimum inhibitory concentration (MIC) patterns of drug-resistant *Mycobacterium tuberculosis* (*M. tuberculosis*) isolates in a retrospective cohort of patients with culture-confirmed DR-TB from August 2019 to July 2023.

**Methods:**

A total of 402 drug-resistant *M. tuberculosis* isolates from clinical patients were included in this study for the analysis of MIC distributions and resistance trends over time. The study included four DR-TB categories: isoniazid-resistant TB, RR-TB, MDR-TB, and pre-XDR-TB. No XDR-TB cases were identified. The temporal distribution of cases was analyzed for seasonal trends, and MIC values were assessed for anti-tuberculosis drugs. A longitudinal analysis was conducted to observe variations in MICs over time. Specifically, a longitudinal comparison was performed on paired *M. tuberculosis* isolates from 49 patients, with isolates collected at intervals exceeding 4 weeks.

**Results:**

The majority of patients were male (72.14%), and the most common form of DR-TB was isoniazid-resistant TB (23.14%). The study found significant seasonal variation, with higher DR-TB incidence in winter and spring months, and the temporal distribution was affected by COVID-19. Resistance rates were highest for isoniazid (40.30%), cycloserine (47.76%), and para-aminosalicylic acid (52.24%). Across the DR-TB cohorts, MICs for isoniazid, ethionamide, para-aminosalicylic acid, and amikacin demonstrated a significant upward trend, whereas a declining pattern was observed for ethambutol. The longitudinal comparison of paired *M. tuberculosis* isolates from 49 patients, demonstrated a marked elevation in the MICs for all 12 antimicrobials over the course of treatment.

**Conclusion:**

This study highlights the increasing burden of DR-TB, with increasing MICs and relatively high resistance rates for a few key drugs indicating reduced drug susceptibility over time. The increasing MICs highlight the need for continuous resistance monitoring and enhanced DR-TB control efforts.

## 1 Introduction

Tuberculosis (TB) remains one of the most significant infectious diseases globally, despite considerable efforts in prevention, diagnosis, and treatment. According to the 2025 WHO Global Report on Tuberculosis (TB), in 2024, China ranked fourth among TB burden countries in terms of estimated TB incidence, accounting for 6.5% (696,000 cases) of the global incidence (WHO, 2025). However, it ranked second in terms of estimated rifampicin-resistant tuberculosis (RR-TB) and multidrug-resistant tuberculosis (MDR-TB) incidence, accounting for 7.1% (28,000 cases) of the global incidence, indicating that the burden of drug-resistant TB in China remains a concern (WHO, 2025). Drug-resistant tuberculosis (DR-TB) poses an even greater challenge due to its resistance to the commonly used first-line drugs, including isoniazid and rifampicin, which are the cornerstone of standard TB treatment. The emergence of these resistant strains complicates TB treatment, leading to prolonged illness, higher mortality rates, and increased healthcare costs, highlighting the need for effective monitoring, prevention, and novel treatment strategies (Farhat et al., 2024).

Minimum inhibitory concentration (MIC) testing offers a quantitative evaluation that strengthens both the accuracy and the clinical utility of drug-resistance assessment in tuberculosis (Mansjo et al., 2025). By measuring the precise level of growth inhibition rather than assigning isolates to broad categorical thresholds, MICs provide a clearer picture of resistance magnitude, something that genotypic methods alone cannot always capture. This quantitative detail also helps resolve discrepancies between genotypic and phenotypic results, particularly for resistance mechanisms whose MIC distributions fall on opposite sides of a clinical breakpoint, such as the gyrA D94G mutation associated with moxifloxacin (Schon et al., 2019). In addition, reliable MIC values improve consistency across laboratories, making it easier to detect systematic errors and reducing the inter-laboratory variability that has been reported for drugs like amikacin, bedaquiline, and linezolid (Rancoita et al., 2018). Ultimately, these advantages make MIC data indispensable for pharmacokinetic/pharmacodynamic-informed adaptive dosing, where accurate estimates of drug activity are fundamental to optimizing individualized treatment regimens (Heysell et al., 2023).

Although MIC testing has emerged as an important approach for quantifying antimicrobial resistance, current evidence is largely restricted to cross-sectional analyses of MIC distributions or studies examining associations between MIC values and resistance-conferring mutations. Some longitudinal studies have shown that resistance-associated mutations can arise during extended treatment and are frequently accompanied by substantial increases in drug MICs (Perumal et al., 2023; Xu et al., 2025). For example, Perumal et al. observed that two of five patients with drug-resistant tuberculosis developed 16- to 64-fold increases in the MICs of ofloxacin and moxifloxacin following the emergence of gyrA D94G/N and A90V mutations during therapy (Perumal et al., 2023). Nevertheless, whether and how MICs change over time at the population level among patients undergoing long-term treatment for drug-resistant tuberculosis remains unclear. Addressing this knowledge gap may improve the clinical interpretation of phenotypic drug susceptibility testing and generate evidence to support more effective treatment monitoring and therapeutic decision-making.

However, there are few studies on MICs of *M. tuberculosis*. In this study, we analyzed the epidemiological characteristics of drug- tuberculosis and determined the MICs of drug-resistant *M. tuberculosis* in low-prevalence areas of tuberculosis in a city in eastern China, and analyzed the distribution and change trend of MICs, which provided a reference basis for the formulation of prevention and control strategies for DR-TB.

## 2 Materials and methods

### 2.1 Isolation and culture of clinical *M. tuberculosis* isolates

In this retrospective observational cohort study, a total of 855 clinical *M. tuberculosis* isolates were isolated from clinical patients in the Fifth People’s Hospital of Wuxi from August 2019 and July 2023. Among these, 458 drug-resistant isolates were identified. To avoid duplicate sampling, only the first drug-resistant isolate from each patient was retained. Consequently, 402 non-duplicate drug-resistant isolates were included in this study for the analysis of MIC distributions and resistance trends over time. Notably, the Fifth People’s Hospital of Wuxi serves as the sole medical institution for conducting drug susceptibility testing for *M. tuberculosis* in Wuxi, Jiangsu Province, China, and all drug-resistant *M. tuberculosis* isolates were included in the study during the research period, therefore, the isolates obtained in this study are considered representative of the drug resistance patterns of drug-resistant *M. tuberculosis* in Wuxi. The tuberculosis incidence rate in Wuxi has dropped to about 20 per 100,000 in recent years, which is considered a low-epidemic area in China. Bronchoalveolar lavage fluid and N-acetyl L-cysteine-2% sodium hydroxide (NALC-NaOH)-digested sputum were cultured using mycobacterial growth indicator tube (MGIT) liquid culture and Löwenstein-Jensen solid medium at 37 °C.

### 2.2 MIC determination

MIC values were determined using the Sensititre™ MYCOTBI plate (Thermo Fisher Scientific USA) according to the manufacturer’s instructions. The following anti-tuberculosis drugs were tested: rifampin, isoniazid, ethambutol, streptomycin, rifabutin, ethionamide, kanamycin, para-aminosalicylic acid, ofloxacin, cycloserine, moxifloxacin, amikacin. The MIC results were interpreted according to the breakpoints and interpretive criteria recommended by the Clinical and Laboratory Standards Institute (CLSI) M24-A2 guideline. The distribution of MIC values was used to assess the level of resistance in the study cohort.

### 2.3 Demographic and clinical data collection

Demographic data, including age, gender, were obtained from the Tuberculosis Information Management System. Isoniazid-resistant TB was defined as resistance to isoniazid and susceptibility to rifampicin. Rifampicin-resistant TB (RR-TB) was defined as resistance to rifampicin, with or without concurrent resistance to other drugs. Multidrug-resistant TB (MDR-TB) was defined as resistance to at least both rifampicin and isoniazid. Pre-XDR-TB was defined as TB that is resistant to rifampicin and any fluoroquinolone. No XDR-TB cases were identified in this study. The distribution of DR-TB cases across different demographic groups was analyzed to identify any significant trends or associations.

### 2.4 Temporal and seasonal distribution analysis

The temporal distribution of DR-TB cases was analyzed by month over the study period from August 2019 to July 2023. Seasonal trends were identified by evaluating the monthly number of DR-TB cases and comparing case counts during the winter (December to February), spring (March to May), summer (June to August), and autumn (September to November) months. The potential impact of COVID-19-related control measures on temporal trends in DR-TB notifications was analyzed using an interrupted time series analysis.

### 2.5 Longitudinal analysis of MIC variations

Among the 855 clinical *M. tuberculosis* isolates, only patients with at least two MIC measurements obtained from isolates collected more than 4 weeks apart and with at least one drug-resistant isolate were eligible for longitudinal analysis. Two isolates from each eligible patient were included, yielding 49 isolate pairs for the analysis of longitudinal MIC changes. Paired MIC measurements from two time points were compared to assess temporal trends in MIC values of *M. tuberculosis* isolates from patients with DR-TB during prolonged treatment.

### 2.6 Statistical analysis

Data were analyzed using descriptive statistics for categorical variables, including frequencies, and proportion, and comparisons between different groups were performed using Fisher’s exact test. The interrupted time-series analysis was performed using segmented negative binomial regression. The Jonckheere-Terpstra test was used to analyze MICs changing trend from 2019 to 2023. Longitudinal changes in MIC values within patients were assessed using the Wilcoxon matched-pairs signed rank test. For multiple comparisons involving the Jonckheere–Terpstra test and Wilcoxon matched-pairs signed rank test, *P*-values were adjusted using the Holm–Bonferroni correction. Statistical significance was set at a *P*-value < 0.05 for all tests. All statistical analyses were performed using GraphPad Prism and SPSS version 26.

## 3 Results

### 3.1 Demographic characteristics of DR-TB cases

As shown in **Table 1**, a total of 402 drug-resistant *M. tuberculosis* isolates were isolated from clinical patients, including 290 males (72.14%) and 112 females (27.86%), with a sex ratio of 2.59:1. The mean age of patients was 53.75 years, ranging from 12 to 92 years. According to the classification definition of DR-TB, 97 (23.14%) were isoniazid-resistant TB, 41 (10.04%) were RR-TB, 38 (9.17%) were MDR-TB, and 52(16.38%) were pre-XDR-TB. These DR-TB cases were mainly distributed in the ≥60 (43.28%) and 40-59 (31.09%) age groups, and were predominantly male (72.14%). The results of the influencing factor analysis showed that the distribution of different types of DR-TB in the population is not affected by different age groups and different genders (all *P* > 0.05).

**Table 1:** The distribution of DR-TB cases with different characteristics in this study.

Characteristics	Isoniazid-resistant TB^*^	RR-TB^*^	MDR-TB^**^	Pre-XDR-TB	Others	Total	Fisher’s exact test
Age group (years)							*P=*0.408
0-19	3	1	2	1	10	17 (4.23%)	
20-39	18	16	6	13	33	86 (21.39)	
40-59	34	11	13	15	52	125 (31.09%)	
≥60	42	13	17	23	79	174 (43.28%)	
Gender							*P=*0.224
Male	64	31	24	37	134	290 (72.14%)	
Female	33	10	14	15	40	112 (27.86%)	
Total	97	41	38	52	174	402	

*Exclude MDR-TB and pre-XDR-TB. **Exclude pre-XDR-TB.

### 3.2 Temporal distribution of DR-TB cases

**Figure 1:**
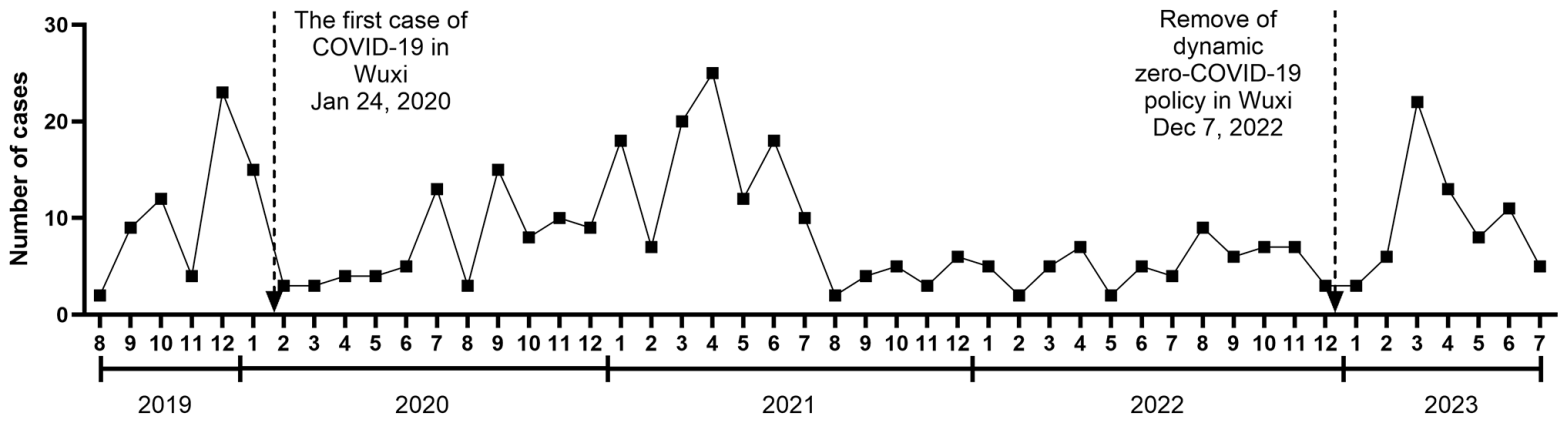
Monthly distribution of the number of DR-TB cases.

The cases of DR-TB were presented monthly from August 2019 to July 2023. The number of cases peaked in April 2021 and then began to decline, reaching a lower level in August 2021 and has remained at a lower level than before. The data in **Figure 1** suggested a potential seasonal pattern. Higher occurrences are observed during the spring (especially March-April) and winter months (especially December-January), while the summer months (especially June-August) generally show lower counts. For instance, December 2019, January 2021, March-April 2021, and April 2022 exhibit increased counts, whereas August 2020 and August 2021 show relatively low occurrences. However, there was no expected peak in the spring of 2020 (March-May), which may be related to the closure and control policy due to COVID-19. Another thing worth noting is that the interrupted time-series analysis demonstrated a significant declining trend in DR-TB cases during the COVID-19 control period (β = -0.412, *P* = 0.044).

### 3.3 MIC distributions of DR-*M. tuberculosis*

**Figure 2:**
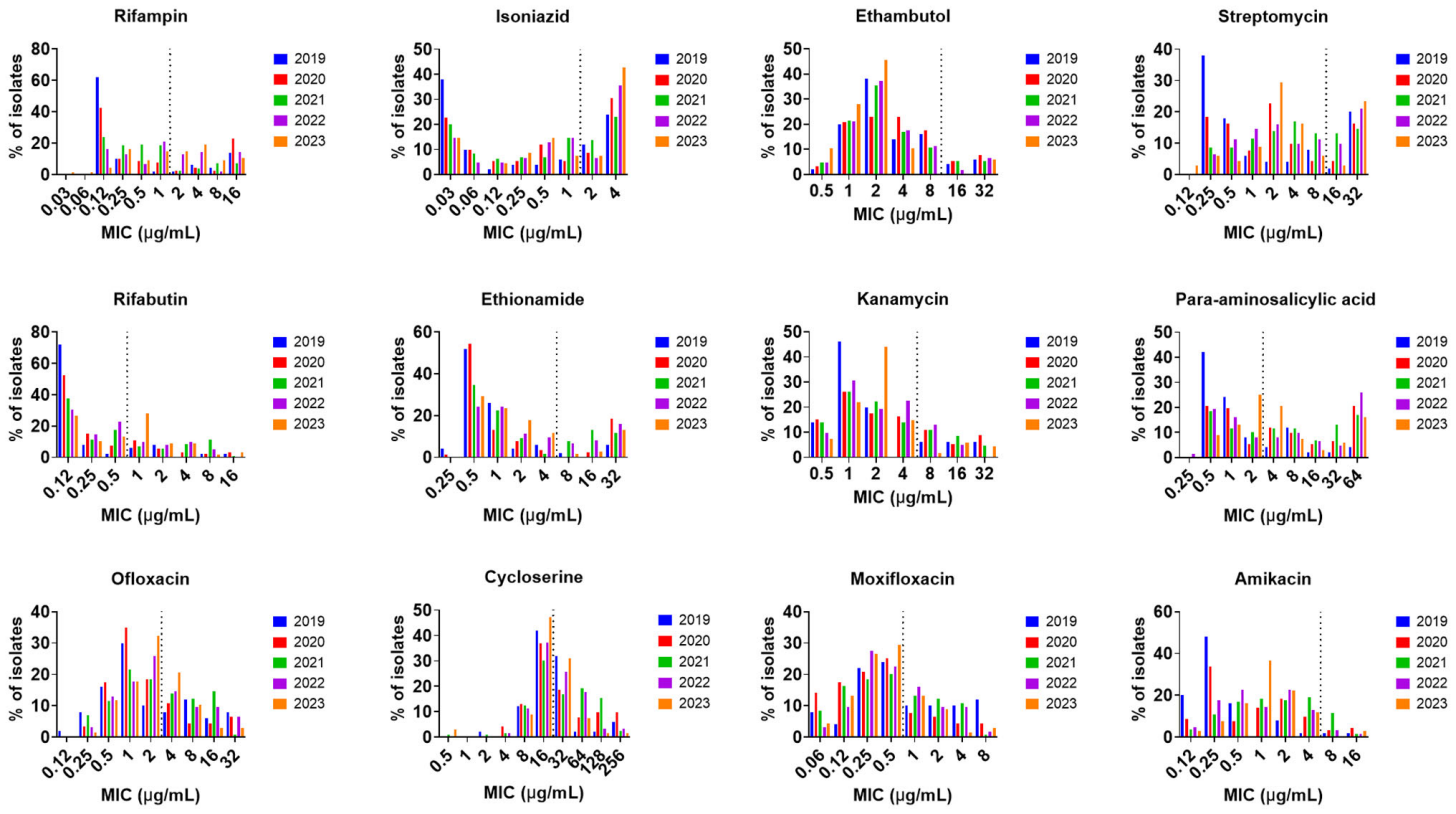
Distribution of MICs among DR-*M. tuberculosis* isolates. The percentages of DR-*M. tuberculosis* isolates at each antimicrobials MIC value (µg/mL) for five consecutive years: 2019 (blue), 2020 (red), 2021 (green), 2022 (purple), and 2023 (orange). The vertical dotted line marks the established clinical breakpoint for antimicrobials susceptibility, separating susceptible (left) from resistant (right) populations.

Amikacin (31/402), ethambutol (40/402) and kanamycin (82/402) were the three anti-tuberculosis drugs with the lowest resistance rates, while para-aminosalicylic acid (210/402), cycloserine (192/402) and isoniazid (162/402) were the three drugs with the highest resistance rates (**Figure 2**). As shown in **Table 2**, from 2019 to 2023, the MICs of rifampin, isoniazid, streptomycin, rifabutin, ethionamide, para-aminosalicylic acid, and amikacin showed a significant increasing trend (all *P* < 0.05), whereas ethambutol showed a significant decreasing trend (*P* < 0.001). No significant temporal changes were observed for kanamycin, ofloxacin, cycloserine, or moxifloxacin.

**Table 2:** Trends in the MICs of DR-*M. tuberculosis* isolates from 2019 to 2023.

Drugs	Year	DR-*M. tuberculosis*				MDR/RR-*M. tuberculosis*			
		MIC_50_ (µg/mL)	MIC_90_ (µg/mL)	*Z*	*P*	MIC_50_ (µg/mL)	MIC_90_ (µg/mL)	*Z*	*P*
Rifampin	2019	0.12	16	5.529	< 0.001	16	16	-4.286	< 0.001
	2020	0.25	16			16	16		
	2021	0.5	8			8	16		
	2022	1	16			4	16		
	2023	2	16			4	16		
Isoniazid	2019	0.12	4	3.238	0.007	2	4	2.043	0.370
	2020	0.5	4			0.5	4		
	2021	1	4			1	4		
	2022	1	4			1	4		
	2023	1	4			4	4		
Ethambutol	2019	2	8	-3.622	0.002	8	32	-4.050	< 0.001
	2020	4	16			4	16		
	2021	2	16			2	16		
	2022	2	8			2	8		
	2023	2	4			2	4		
Streptomycin	2019	0.5	32	3.903	< 0.001	32	32	0.853	1.000
	2020	2	32			1	32		
	2021	4	32			16	32		
	2022	4	32			8	32		
	2023	2	32			4	32		
Rifabutin	2019	0.12	2	5.129	< 0.001	1	2	0.800	1.000
	2020	0.12	2			1	8		
	2021	0.5	8			2	8		
	2022	0.5	4			1	8		
	2023	0.5	4			1	4		
Ethionamide	2019	0.5	4	4.102	< 0.001	1	32	1.676	0.563
	2020	0.5	32			0.5	32		
	2021	1	32			2	16		
	2022	2	32			2	32		
	2023	1	32			2	32		
Kanamycin	2019	1	16	1.330	0.551	1	16	1.404	0.081
	2020	2	16			2	32		
	2021	2	16			2	8		
	2022	2	8			2	8		
	2023	2	16			2	4		
Para-aminosalicylic acid	2019	1	8	3.137	0.009	1	64	1.750	0.560
	2020	4	64			1	64		
	2021	4	64			4	64		
	2022	4	64			8	64		
	2023	4	64			2	64		
Ofloxacin	2019	1	16	2.355	0.074	4	16	1.988	0.374
	2020	1	16			1	16		
	2021	2	16			4	16		
	2022	2	16			4	16		
	2023	2	8			2	16		
Cycloserine	2019	16	32	-0.248	1.000	16	128	1.217	0.895
	2020	16	128			16	256		
	2021	32	128			32	128		
	2022	16	64			16	64		
	2023	16	64			32	64		
Moxifloxacin	2019	0.5	8	-0.009	1.000	1	4	-0.775	1.000
	2020	0.25	2			0.5	8		
	2021	0.5	4			0.5	4		
	2022	0.5	4			0.5	2		
	2023	0.5	2			0.5	2		
Amikacin	2019	0.25	2	4.879	< 0.001	0.25	4	4.625	< 0.001
	2020	0.5	4			0.25	16		
	2021	1	8			1	8		
	2022	1	4			1	8		
	2023	1	4			1	4		

### 3.4 MIC distributions of MDR/RR-*M. tuberculosis*

**Figure 3:**
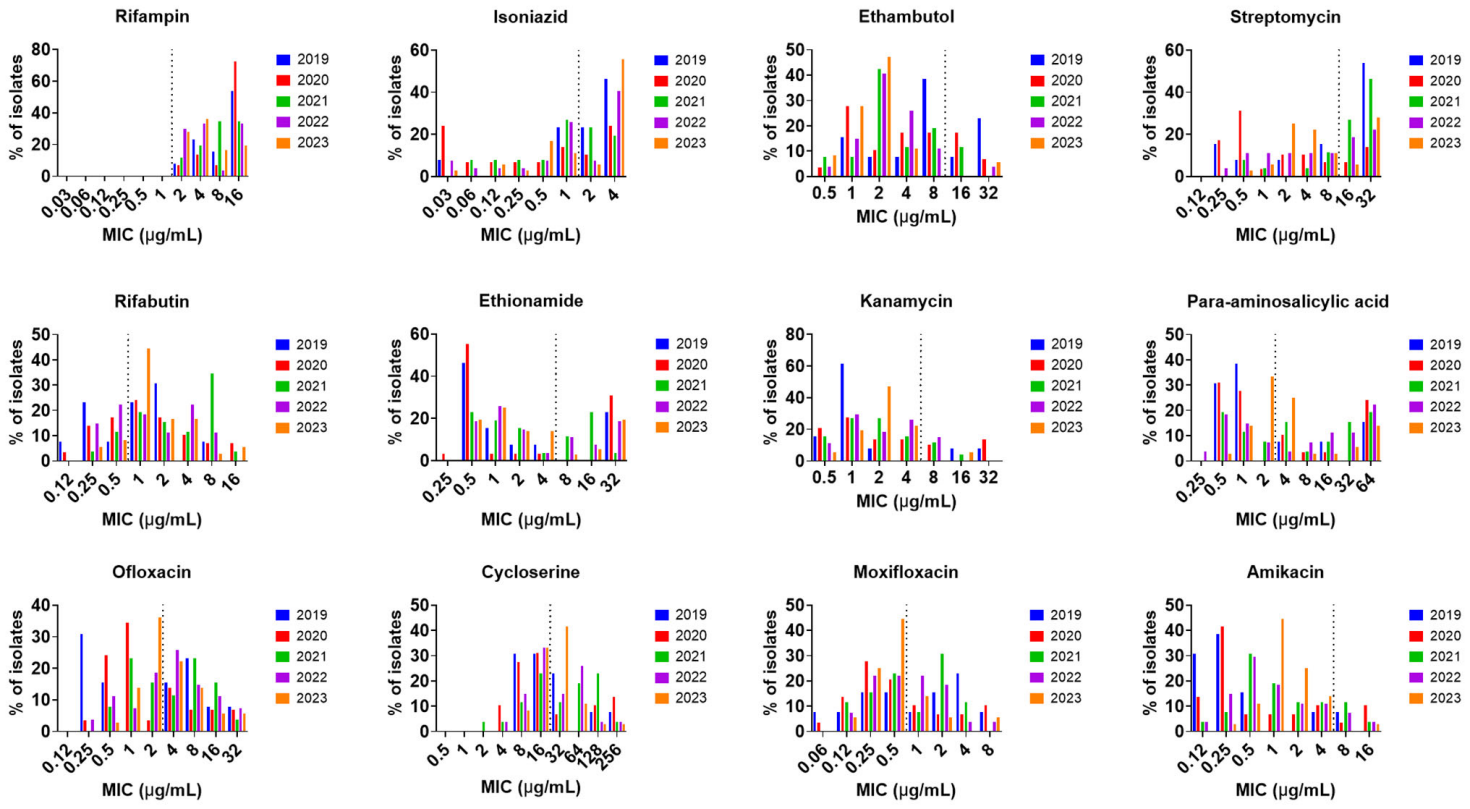
Distribution of MICs among MDR/RR-*M. tuberculosis* isolates. The percentages of MDR/RR -*M. tuberculosis* isolates at each antimicrobials MIC value (µg/mL) for five consecutive years: 2019 (blue), 2020 (red), 2021 (green), 2022 (purple), and 2023 (orange). The vertical dotted line marks the established clinical breakpoint for antimicrobials susceptibility, separating susceptible (left) from resistant (right) populations.

Given that MDR/RR-TB is the most concerning DR-TB, the MIC distributions of MDR/RR-*M. tuberculosis* were analyzed. Except for rifampicin, which is a must-have anti-tuberculosis drug for MDR/RR-TB, the anti-tuberculosis drug with the highest resistance rate was rifabutin (97/131), followed by isoniazid (65/131), and para-aminosalicylic acid (65/131), ofloxacin (64/131). The three anti-tuberculosis drugs with the lowest resistance rates are amikacin (13/131), ethambutol (17/131) and kanamycin (19/131)(**Figure 3**). From 2019 to 2023, a significant increasing trend in MIC values was observed for amikacin (*P* < 0.05)(**Table 2**). In contrast, rifampicin and ethambutol exhibited significant decreasing trends in MICs (all *P* < 0.001), while no significant temporal trends were detected for other anti-tuberculosis drugs.

### 3.5 Longitudinal changes in MIC values of *M. tuberculosis* isolates during prolonged treatment of DR-TB

**Figure 4:**
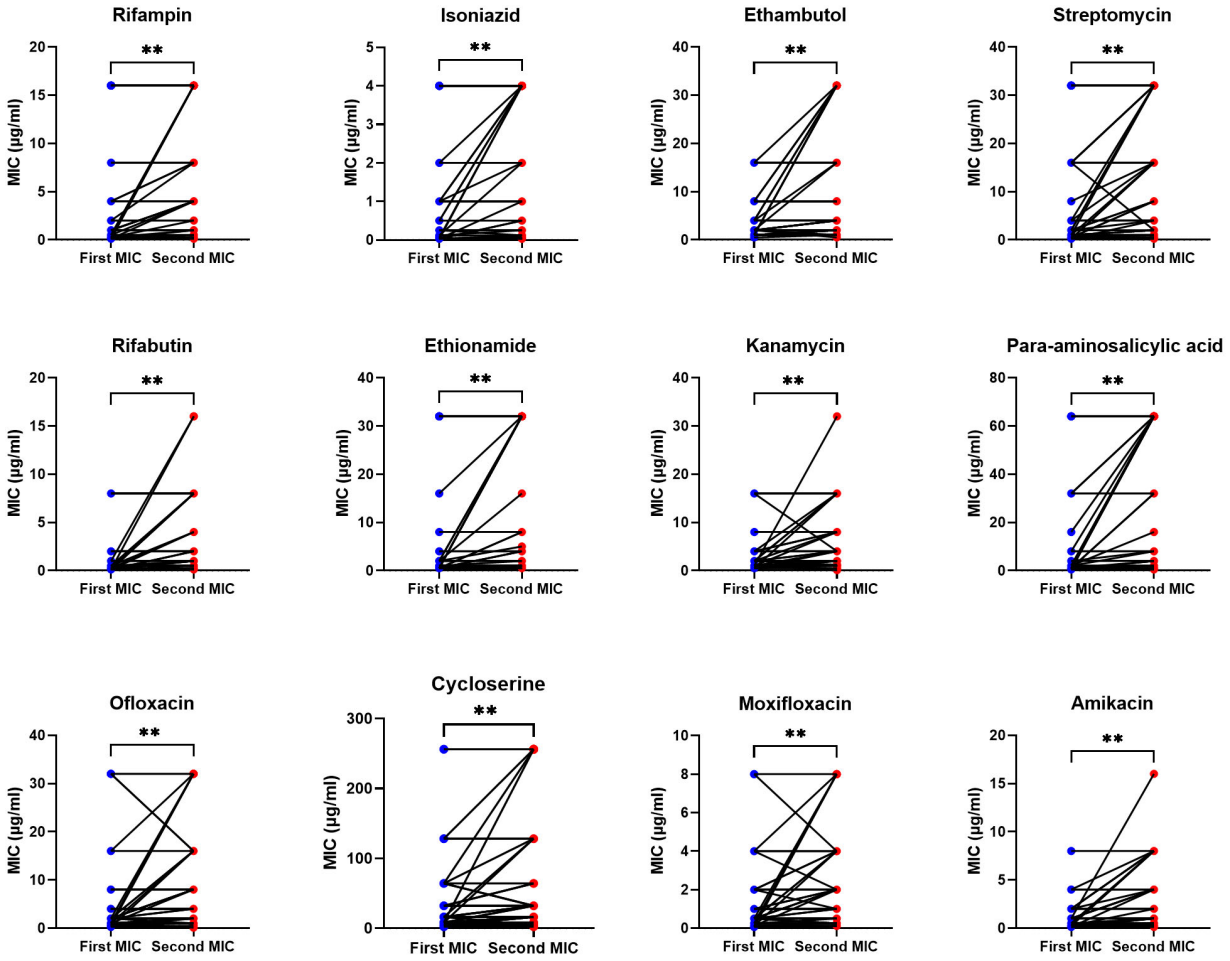
Changes in MIC values of DR-*M. tuberculosis*. **P < 0.01.

To investigate the potential changes in MIC values of *M. tuberculosis* isolates from patients with DR-TB during prolonged treatment, we conducted a longitudinal analysis of MIC values from two bacterial isolates per patient, collected from 49 patients at intervals exceeding 4 weeks. The paired-sampling interval as a median of 157 (IQR, 70-314) days. The results shown in **Figure 4** indicated that the MICs of the 12 anti-tuberculosis drugs measured were significantly increased. Unexpectedly, decreased MIC values for streptomycin, kanamycin, ofloxacin, and moxifloxacin were also demonstrated by several *M. tuberculosis* isolates. A total of 588 paired MIC values across 12 anti-tuberculosis drugs were analyzed from 49 patients. Of these, 303 (51.5%) remained unchanged, while 259 (44.0%) and 26 (4.4%) exhibited increased and decreased MICs, respectively. Notably, 132 (22.4%) MIC increases and 2 (0.3%) MIC decreases crossed the critical concentration threshold. Among the 49 cases, 7 initially non-drug-resistant cases progressed to DR-TB cases, 3 isoniazid-resistant TB cases progressed to MDR or pre-XDR-TB cases, 3 RR-TB cases progressed to MDR or pre-XDR-TB cases, while 5 other TB cases developed heterogeneously into isoniazid-resistant TB or pre-XDR-TB cases, indicating variable patterns of resistance progression.

## 4 Discussion

This study identified a total of 402 DR-TB cases, predominantly male, with an overall mean age of 53.75 years, of which 23.14% were classified as isoniazid-resistant TB. The study also revealed that there was no significant difference in DR-TB distribution between different age groups or genders. The temporal distribution of drug-resistant TB cases exhibited seasonal fluctuations, with peaks in the winter and spring months, and the temporal distribution has been affected by COVID-19. From 2019 to 2023, among the DR-TB cohorts, the MICs of isoniazid, ethionamide, para-aminosalicylic acid, and amikacin showed an increasing trend, whereas ethambutol exhibited a decreasing trend. Longitudinal analysis of two *M. tuberculosis* isolates per patient over intervals exceeding 4 weeks revealed a significant increase in the MICs of all 12 tested anti-tuberculosis drugs during the treatment.

The male predominance and middle-to-older age distribution observed in this study are consistent with global DR-TB patterns, where males are more commonly affected owing to greater exposure to behavioral risk factors, and older adults are more susceptible because of immunosenescence and cumulative TB exposure (Oladimeji et al., 2023; Chopra et al., 2022). Notably, gender and age were not significantly associated with DR-TB in our analysis, whereas other studies have identified irregular treatment adherence and previous TB history as the main drivers of MDR-TB (Receado et al., 2025).

The spring–winter peak in DR-TB cases parallels findings from several studies and may be attributable to delayed diagnosis, increased indoor crowding, and weakened immunity during colder months (Lin and Liao, 2014; Li et al., 2019). However, an autumn peak reported in Guizhou, China (Yun et al., 2022), suggests that seasonal patterns are likely region-specific, shaped by healthcare capacity, socioeconomic factors, demographics, and meteorological conditions (Li et al., 2019).

The post-2021 decline in DR-TB notifications, which coincided with the enforcement of COVID-19 restrictions, mirrors global decreases in TB case detection caused by lockdowns and healthcare resource diversion (Tovar et al., 2022). Although such measures may have transiently reduced TB transmission, their long-term impact on DR-TB dynamics remains uncertain. Indeed, the COVID-19 pandemic has generally disrupted DR-TB control and increased cases, yet in some settings the associated containment measures might have unintentionally suppressed TB spread (Silva et al., 2023; Zouganeli et al., 2025).

The study revealed significant resistance to several first-line and second-line drugs in the DR-TB isolates, with isoniazid, cycloserine, and para-aminosalicylic acid exhibiting the highest resistance rates. These findings align with global trends indicating that first- and second--line drugs continue to show high resistance rates in DR-TB populations (Luo et al., 2019; Wu et al., 2022; Jin et al., 2024). However, rifabutin exhibited the highest resistance rate among MDR/RR-TB isolates, this observation can be largely attributed to the pronounced cross-resistance between rifabutin and rifampicin, as both agents belong to the rifamycin class and share the same molecular target. Given that rifampicin resistance is a defining feature of MDR/RR-TB, resistance to rifabutin is therefore expected to be highly prevalent in these strains. A high level of ofloxacin resistance was observed among MDR/RR-TB patients in this study, in line with prior RR-TB data reporting substantial resistance to fluoroquinolones, including levofloxacin (26.1%) (Teng et al., 2024). The higher resistance observed here may reflect drug-specific differences, as ofloxacin is a less potent, earlier-generation fluoroquinolone with more extensive historical use, which may predispose to higher resistance prevalence. These findings are similar to some Chinese results but different results have been seen globally, such as a lower rate of resistance to para-aminosalicylic in multidrug-resistant tuberculosis in a multicenter study in South Korea, which may indicate geographical differences in tuberculosis drug resistance (Lee et al., 2021).

Although some studies have investigated the MICs of *M. tuberculosis*, most have been cross-sectional in design (Burke et al., 2017; Getahun et al., 2022; Guo et al., 2023). In contrast, the present study reveals distinct temporal dynamics in MIC distributions among drug-resistant *M. tuberculosis* isolates in a low-epidemic region of China from 2019 to 2023. Among DR-TB isolates, significant upward shifts in MICs for multiple first- and second-line agents—including isoniazid, fluoroquinolones, aminoglycosides, and thioamides—may reflect a trend toward reduced drug susceptibility over time, although there may be no categorical transitions across critical concentrations. Such MIC creep likely reflects cumulative selective pressure from prolonged and repeated drug exposure, suboptimal treatment regimens, and heterogeneous pharmacokinetics, which together favor the enrichment of mutations conferring incremental reductions in susceptibility (Jones et al., 2022). In this context, the simultaneous increase in MICs of drugs targeting different targets warrants further attention. It is noteworthy that some anti-tuberculosis drugs, such as ethambutol, showed a decrease in MIC values in this study, which may be related to changes in the medication regimen. In Nanjing, another city in Jiangsu Province, China, which is very close to Wuxi, an analysis of tuberculosis drug utilization from 2019 to 2024 revealed that ethambutol exhibited the largest decrease in defined daily doses (DDDs), dropping from the second to the seventh rank (Pan, 2025). Consequently, ethambutol-resistant strains that once dominated under high ethambutol exposure may be gradually outcompeted by more susceptible strains that regain fitness in the absence of ethambutol selection. However, other factors, such as spontaneous genetic reversion, compensatory mutations, or shifts in bacterial population dynamics, could also contribute to the observed MIC reduction. These findings underscore the need for more potent drug combinations and highlight the critical importance of timely and accurate drug susceptibility testing for all TB patients, particularly those with a history of prior treatment or an elevated risk of drug resistance.

The longitudinal analysis in this study, which tracked MIC variations, reveals important trends in the increasing resistance of *M. tuberculosis* isolates. In the absence of evidence for reinfection with a different M. tuberculosis strain, the observed drug resistance most plausibly suggests potential intra-host evolution of resistance. This process was likely driven by insufficient drug pressure, resulting from sustained selective pressure under subtherapeutic drug concentrations or incomplete treatment courses. A previous study on intra-host evolution of *M. tuberculosis* among patients with DR-TB demonstrated that the MICs of *M. tuberculosis* can increase within two months of treatment, with drug-resistance–associated mutations even emerging one month during the treatment (Perumal et al., 2023). Based on these findings, the time interval in the present study was set to four weeks or longer to ensure that potential changes in MICs were not overlooked.

The MICs of most anti-tuberculosis drugs were lower in newly treated patients and in those with a single prior treatment episode than in retreated patients or those with multiple treatment episodes of MDR-TB (Tang et al., 2022). These findings indirectly suggest that MICs tend to increase with repeated or prolonged tuberculosis treatment. This phenomenon suggests that not only does resistance develop over time, but it can also result from inadequate or inappropriate drug use. The findings of this study, in which some isolates exhibited reduced MIC values for antimicrobials such as streptomycin, kanamycin, ofloxacin, and moxifloxacin, may indicate a potential restoration of susceptibility or a shift in resistance profiles to these agents. However, such changes need careful monitoring to determine whether they reflect true reversals in resistance or simply fluctuations due to the heterogeneous nature of TB populations.

One of the limitations of this study is the relatively small sample size of 402 isolates, which may not fully capture the diversity of DR-TB strains present in larger and more heterogeneous populations. In addition, this study does not encompass all drug-resistant tuberculosis cases, as some *M. tuberculosis* isolates may not have been successfully cultured, and certain cases may remain undiagnosed or not present for medical evaluation. As a hospital-based retrospective study, referral bias, case-mix differences, and the restriction of the study population to specific geographic and clinical settings may have influenced the observed resistance patterns and limited the generalizability of the findings. Furthermore, because this study was primarily based on microbiological surveillance data, detailed clinical information, including prior treatment exposure, treatment duration, treatment adherence, comorbidities, and pharmacokinetic parameters, was not systematically available, precluding robust multivariable analyses to investigate factors associated with changes in MIC values. Therefore, the observed longitudinal increases in MICs should be interpreted cautiously and not regarded as definitive evidence of resistance amplification alone, as alternative explanations, such as reinfection with genetically distinct strains, intra-host bacterial heterogeneity, laboratory measurement variability, and differences in drug exposure, cannot be excluded. Moreover, because molecular genotyping was not performed, we were unable to differentiate acquired drug resistance from reinfection, mixed infection, or heteroresistance in the 49 patients with longitudinal isolates, nor could we confirm the clonal relatedness of paired isolates. In addition, these 49 patients may represent a selected subgroup of individuals who survived longer and had repeated cultures performed, potentially introducing survivor bias and limiting the representativeness of longitudinal MIC trends. In addition, the absence of more extensive longitudinal follow-up data may have limited the assessment of long-term trends in drug resistance and treatment outcomes. Finally, reliance on phenotypic MIC testing alone may not fully capture the genetic mechanisms underlying resistance, highlighting the need for future studies integrating clinical, microbiological, and genomic data to better elucidate the drivers of MIC variation and resistance evolution in DR-TB.

In conclusion, this study highlights a substantial burden of DR-TB, with the most common form of DR-TB being isoniazid-resistant TB, and no significant differences by age or gender. Seasonal variations and the COVID-19 pandemic influenced case distribution. Increasing MICs for key anti-tuberculosis drugs and relatively high resistance rates among DR isolates may reflect a trend toward reduced drug susceptibility over time. The observed rise in MICs during treatment suggests the potential for within-host resistance amplification, though further genomic studies are required to rule out reinfection, emphasizing the need for continuous resistance monitoring and optimized, individualized treatment strategies.

## Data Availability

The raw data supporting the conclusions of this article will be made available by the authors, without undue reservation.
